# Supplementary material for: Vaccines against the original strain of SARS-CoV-2 provide T cell memory to the B.1.1.529 variant
Source: Commun Med (Lond). 2022 Nov 2;2:140. doi: 10.1038/s43856-022-00203-7 (PMC9629766; doi:10.1038/s43856-022-00203-7)
Supplement: Supplementary file 1 — Supplementary Information [file 43856_2022_203_MOESM1_ESM.pdf]

- Supplementary Information -

Vaccines against the original strain of SARS-CoV-2 provide T cell memory to the B.1.1.529 variant

Charlyn Dörnte[1], Verena Traska[1], Nicole Jansen[1], Julia Kostyra[1], Herrad Baurmann[1], Gereon Lauer[1], Yi-Ju Huang[1], Sven Kramer[1], Olaf Brauns[1], Holger Winkels[2], Jürgen Schmitz[1], Christian Dose[1], Anne Richter[1] and Marc Schuster[1,3]

[1]Miltenyi Biotec B.V. & Co. KG, Friedrich-Ebert-Straße 68, 51429 Bergisch Gladbach, Germany
[2]University of Cologne, Faculty of Medicine and University Hospital Cologne, Clinic III for Internal Medicine, Cologne, Germany
[3]Corresponding Author: marcsch@miltenyi.com

Supplementary Table 1

Supplementary Figure 1

Supplementary Figure 2

Supplementary Figure 3

Supplementary Figure 4

Supplementary Data - Captions

Group	Age distribution	gender distribution	Donor ID	Vaccine received as 1st dose or Natural Infection	Days after 1st vaccination when receiving the 2nd dose	Vaccine received as 2nd dose	Days after 2nd vaccination when receiving the 3rd dose	Vaccine received as 3rd dose	Day of sample collection after 1st vaccination
2x vaccinated	age range: 27 - 54 median age: 42	female: 75%; male: 25%	1	Comirnaty	d41	Comirnaty	-	-	d285
			2	Comirnaty	d28	Comirnaty	-	-	d237
			3	Comirnaty	d22	Comirnaty	-	-	d126
			4	Comirnaty	d40	Comirnaty	-	-	d207
			5	Comirnaty	d43	Comirnaty	-	-	d216
			6	Comirnaty	d42	Comirnaty	-	-	d221
			7	Comirnaty	d45	Comirnaty	-	-	d200
			8	Comirnaty	d21	Comirnaty	-	-	d52
3x vaccinated	age range: 28 - 53 median age: 47	female: 60%; male: 40%	9	Comirnaty	d28	Comirnaty	d198	Spikevax	d264
			10	Comirnaty	d29	Comirnaty	d196	Comirnaty	d271
			11	Comirnaty	d42	Comirnaty	d166	Comirnaty	d241
			12	Natural Infection	d185	Comirnaty	d123	Spikevax	d360
			13	Comirnaty	d32	Comirnaty	d183	Comirnaty	d250
			14	Comirnaty	d28	Comirnaty	d131	Spikevax	d170
			15	Vaxzevria	d48	Comirnaty	d159	Spikevax	d241
			16	Vaxzevria	d46	Comirnaty	d155	Spikevax	d235
			17	Vaxzevria	d77	Comirnaty	d186	Spikevax	d301
			18	Vaxzevria	d52	Comirnaty	d102	Spikevax	d249

Supplementary Table 1: Overview of the age, sex, date of vaccinations and type of vaccines of the study subjects.

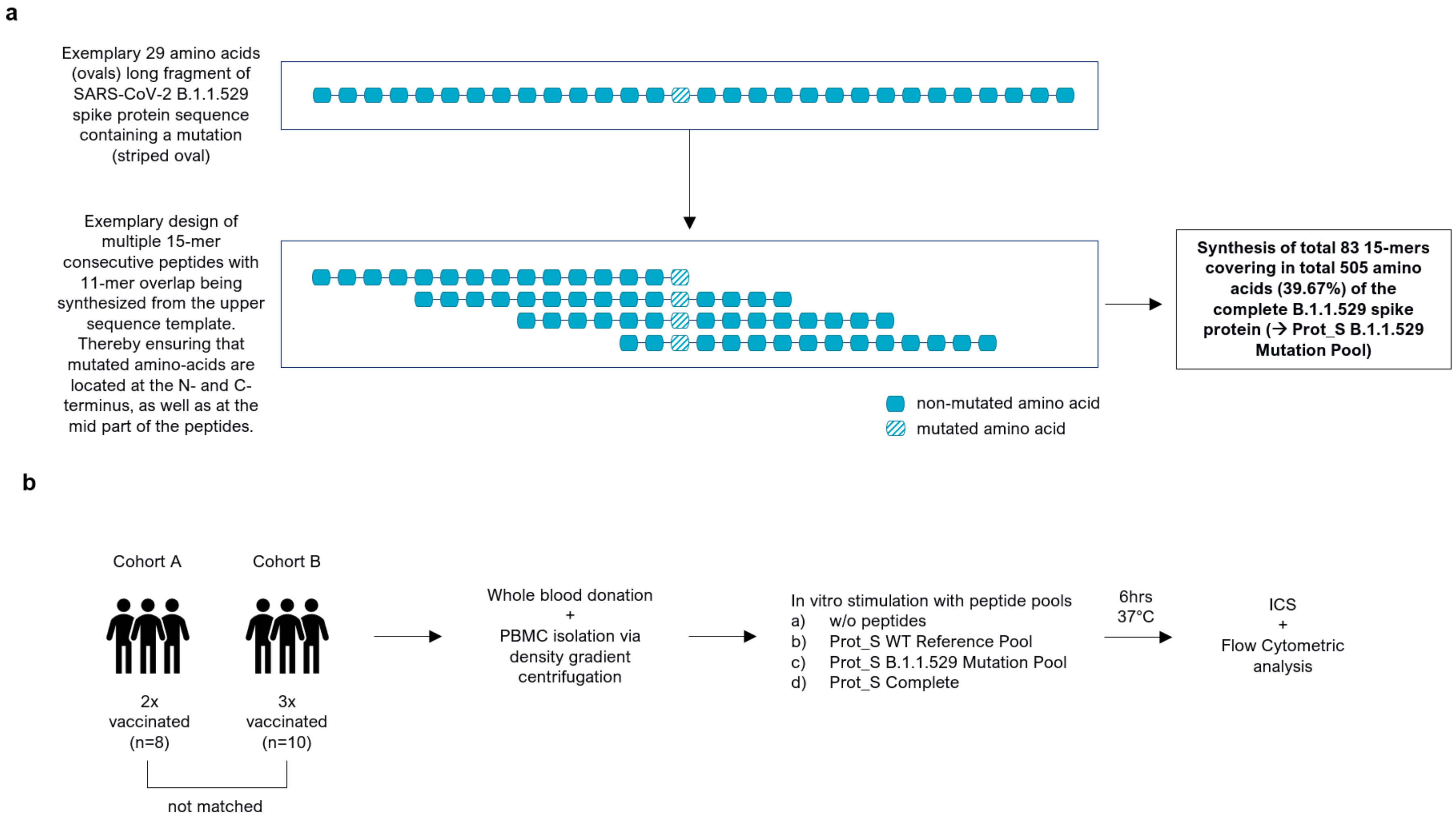
a
Exemplary 29 amino acids (ovals) long fragment of SARS-CoV-2 B.1.1.529 spike protein sequence containing a mutation (striped oval)
Exemplary design of multiple 15-mer consecutive peptides with 11-mer overlap being synthesized from the upper sequence template. Thereby ensuring that mutated amino-acids are located at the N- and C-terminus, as well as at the mid part of the peptides.
Synthesis of total 83 15-mers covering in total 505 amino acids (39.67%) of the complete B.1.1.529 spike protein (→ Prot_S B.1.1.529 Mutation Pool)
non-mutated amino acid
mutated amino acid
b
Cohort A
Cohort B
2x vaccinated (n=8)
3x vaccinated (n=10)
not matched
Whole blood donation + PBMC isolation via density gradient centrifugation
In vitro stimulation with peptide pools
a) w/o peptides
b) Prot_S WT Reference Pool
c) Prot_S B.1.1.529 Mutation Pool
d) Prot_S Complete
6hrs 37°C
ICS + Flow Cytometric analysis

Supplementary Figure 1 Schematic illustration of the peptide design and workflow of the *in vitro* stimulation approach.

(a) Exemplary design of peptides used for *in vitro* stimulations. Using the SARS-CoV-2 spike protein amino acid sequence as a template consecutive 15-mer peptides with 11-mer overlaps were designed. This is exemplary shown for the Prot_S B.1.1.529 mutation pool, which is finally made up of 83 15-mer peptides covering all mutated parts of the B.1.1.529 SARS-CoV-2 spike protein, representing approximately 40% of the complete amino acid sequence. **(b)** Schematic illustration of the experimental workflow. Whole blood donations from both cohorts, 2x and 3x vaccinated individuals were taken and PBMC freshly prepared via density gradient centrifugation. Afterwards T cells were stimulated with three different peptide pools: Prot_S WT Reference Pool, Prot_S B.1.1.529 Mutation Pool, or Prot_S Complete. Additionally a negative control was prepared for each donor. After six hours stimulation the frequency of activated cells was determined via an intracellular cytokine staining (ICS) and flow cytometric analysis.

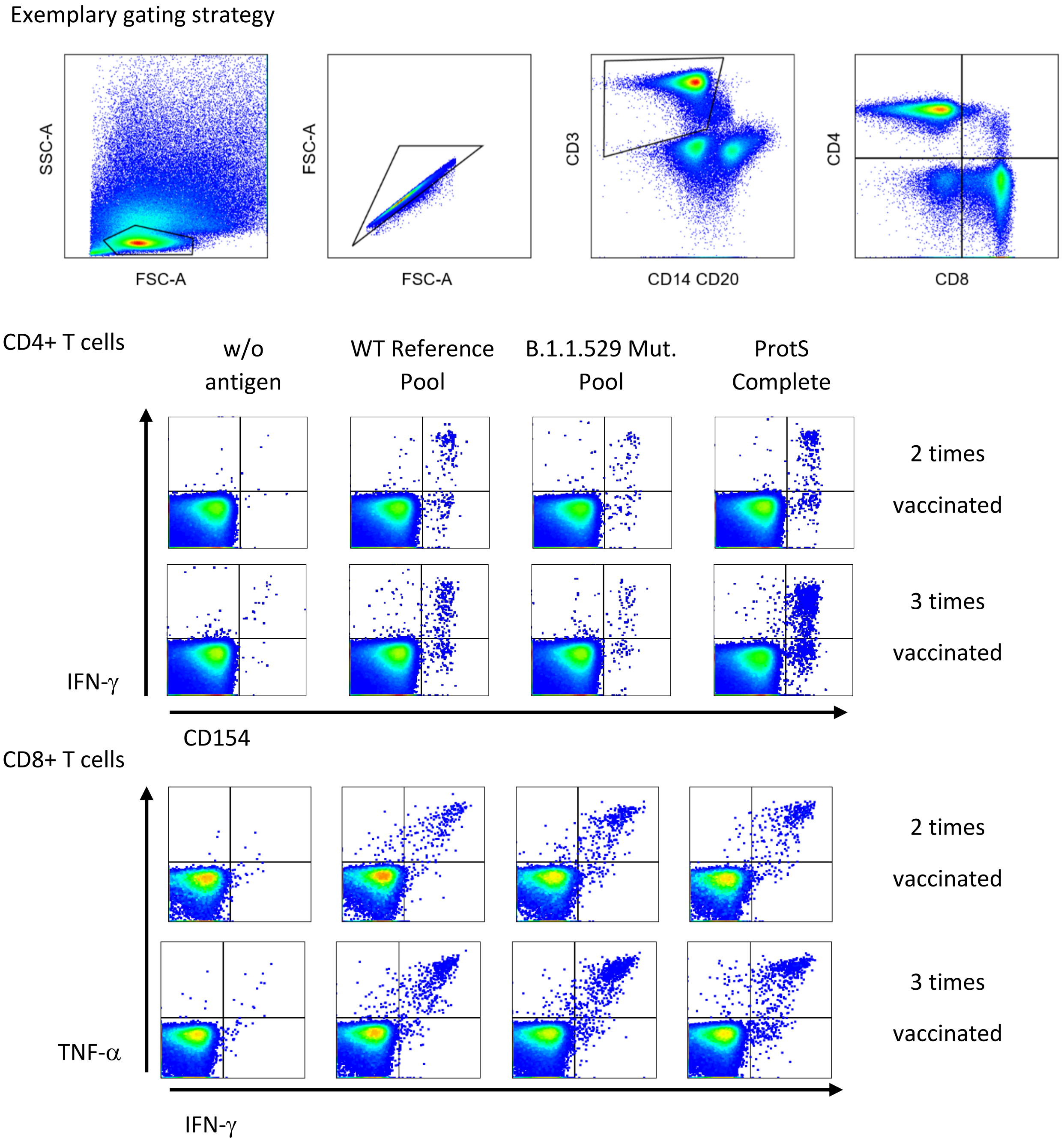

Supplementary Figure 2: Flow analysis of T cell responses upon stimulation with Omicron and reference pools

Exemplary gating strategy and pseudo-color dot plots for the analysis of IFN-γ and CD154 expression within CD4 T cells (top) and of TNF-α and IFN-γ expression among CD8 T cells (bottom) are shown. The labeling of the axes represents the analyzed parameters. Upper plots in each panel show data from 2 times vaccinated subjects and lower plots data from 3 times vaccinated study subjects. On top the negative control and the peptide pool used for stimulation are indicated. For the corresponding analyses in Figure 1, the frequencies of the double positive populations were analyzed

<table>
<tr><td></td><td colspan="2">CD4+ T cell compartment</td></tr>
<tr><td></td><td>Power</td><td>Group Size (n)</td></tr>
<tr><td></td><td>0.8</td><td>10/8 samples / cohort</td></tr>
<tr><td>Power Model</td><td colspan="2">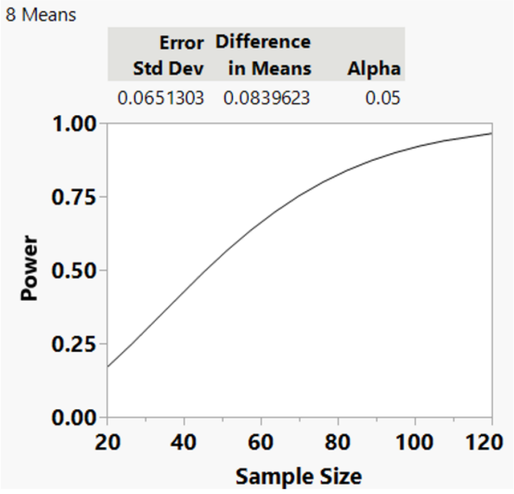
</td></tr>
</table>

Supplementary Figure 3 Analysis of statistical power of in vitro stimulation experiments

Statistical power analyses of the *in vitro* stimulation experiments for CD4+ T cells (right column). The means of all eight measurements detected under CD4+ T cells, as well as the greatest standard deviations have been used for the calculation of the given statistical power. Furthermore, for both calculations the significance threshold α was set to 0.05. The resulting power model (lower row) shows the power (y-axis) that is given under a respective sample size (x-axis).

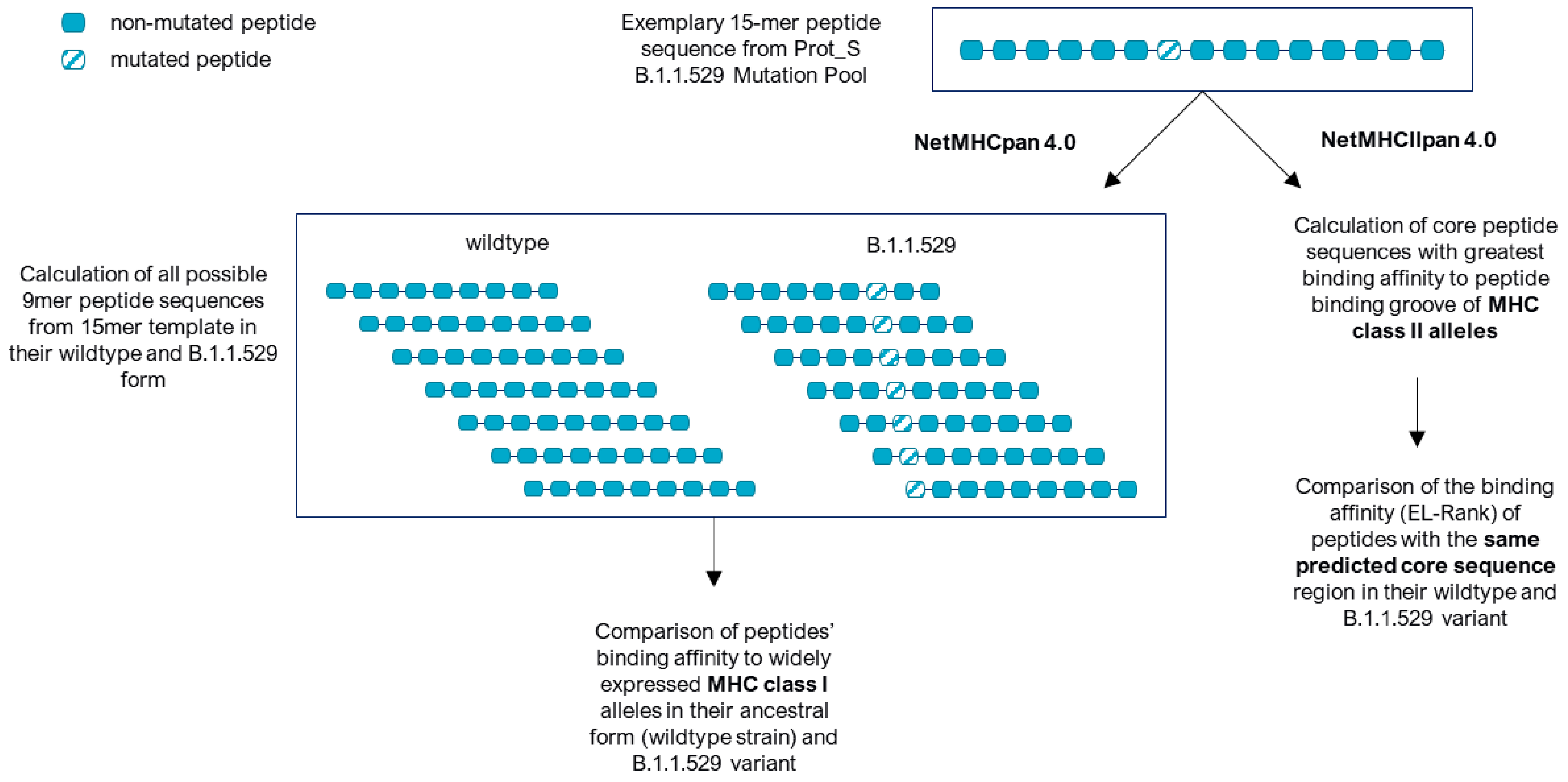

Supplementary Figure 4 ***In Silico*** **approach comparing peptide affinities to MHC class I and MHC class II, respectively, of peptides in their ancestral (wildtype) and mutated (B.1.1.529) version.**

Shown is the schematic workflow of the performed *in silico* NetMHC(II)pan-based determination of the binding affinities (EL-Rank) of peptides in their wildtype and their B.1.1.529 variant. This analysis was performed for the most widely expressed MHC class I and MHC class II allotypes in Europe using the NetMHCpan and the NetMHCIIpan algorithm, respectively. Finally, the determined EL-Ranks were categorized and changes between the wildtype and the B.1.1.529 variant tracked.

Supplementary Data (Excel File)

Sheet 1 Raw Data for figure 1a

Given are the frequencies [%] of CD154+IFN-γ+CD4+ T cells detected upon stimulation with peptide pools (“WT Reference Pool”, “B.1.1.529 Mutation Pool”, and “Prot_S Complete”), or without any stimulatory additive (“w/o antigen”). Each row underneath the two distinct cohorts, “2x vaccinated (n=8)” (left column) and “3x vaccinated (n=10)” (right column), represents one study subject.

Sheet 2 Raw Data for figure 1b

Given are the frequencies [%] of IFNg+ TNFa+ CD8 T cells detected upon stimulation with peptide pools (“WT Reference Pool”, “B.1.1.529 Mutation Pool”, “Prot_S Complete”), or without any stimulatory additive (“w/o antigen”). Each row underneath the two distinct cohorts “2x vaccinated (n=8)” (left column) and “3x vaccinated (n=10)” (right column), represents one study subject.

Sheet 3 Raw Data for Figure 2a

Given are the EL-Ranks (OMI_EL_Rank, WT_EL_Rank) for HLA class II allotypes corresponding to the 9-mer core peptides derived from either the SARS-CoV-2 Omicron variant (Core_Omicron) or the SARS-CoV-2 wildtype strain (Core_WT). These 9mer core peptides originate from corresponding 15-mer peptides (“15mer Peptide Omicron” and “15mer Peptide WT”) (lower table). For each of the analyzed HLA allotypes, the absolute number of peptides with inter-variant differences in their ability to bind to the respective binding groove are given (“# Peptides with improved binding in WT form”; “# Peptides with improved binding in Omicron form”) (upper table).

Sheet 4 Raw Data for Figure 2b

Given are the EL-Ranks (OMI_EL_Rank, WT_EL_Rank) for HLA class I allotypes corresponding to the 222 9-mer peptides (Peptide Sequences) derived from either the SARS-CoV-2 Omicron variant (Omicron Peptide) or the SARS-CoV-2 wildtype strain (WT Peptide) (lower table). Additionally, for each of the analyzed HLA allotypes, the absolute number of peptides with inter-variant differences in their ability to bind to the respective binding groove are given (“# Peptides with improved binding in WT form”; “# Peptides with improved binding in Omicron form”) (upper table).
