# Supplementary material for: Vaccines against the original strain of SARS-CoV-2 provide T cell memory to the B.1.1.529 variant
Source: Commun Med (Lond). 2022 Nov 2;2:140. doi: 10.1038/s43856-022-00203-7 (PMC9629766; doi:10.1038/s43856-022-00203-7)
Supplement: Supplementary file 3 — Description of Additional Supplementary Files [file 43856_2022_203_MOESM3_ESM.pdf]

Description of Additional Supplementary Files

File Name: Supplementary Data 1

Description:

Sheet 1 Raw Data for figure 1a Given are the frequencies [%] of CD154+IFN-γ+CD4+ T cells detected upon stimulation with peptide pools (“WT Reference Pool”, “B.1.1.529 Mutation Pool”, and “Prot_S Complete”), or without any stimulatory additive (“w/o antigen”). Each row underneath the two distinct cohorts, “2x vaccinated (n=8)” (left column) and “3x vaccinated (n=10)” (right column), represents one study subject.

Sheet 2 Raw Data for figure 1b Given are the frequencies [%] of IFNg+ TNFa+ CD8 T cells detected upon stimulation with peptide pools (“WT Reference Pool”, “B.1.1.529 Mutation Pool”, “Prot_S Complete”), or without any stimulatory additive (“w/o antigen”). Each row underneath the two distinct cohorts “2x vaccinated (n=8)” (left column) and “3x vaccinated (n=10)” (right column), represents one study subject.

Sheet 3 Raw Data for Figure 2a
Given are the EL-Ranks (OMI_EL_Rank, WT_EL_Rank) for HLA class II allotypes corresponding to the 9-mer core peptides derived from either the SARS-CoV-2 Omicron variant (Core_Omicron) or the SARSCoV-2 wildtype strain (Core_WT). These 9mer core peptides originate from corresponding 15-mer peptides (“15mer Peptide Omicron” and “15mer Peptide WT”) (lower table). For each of the analyzedHLA allotypes, the absolute number of peptides with inter-variant differences in their ability to bind to the respective binding groove are given (“# Peptides with improved binding in WT form”; “# Peptides with improved binding in Omicron form”) (upper table).

Sheet 4 Raw Data for Figure 2b
Given are the EL-Ranks (OMI_EL_Rank, WT_EL_Rank) for HLA class I allotypes corresponding to the 222 9-mer peptides (Peptide Sequences) derived from either the SARS-CoV-2 Omicron variant (Omicron Peptide) or the SARS-CoV-2 wildtype strain (WT Peptide) (lower table). Additionally, for each of the analyzed HLA allotypes, the absolute number of peptides with inter-variant differences in their ability to bind to the respective binding groove are given (“# Peptides with improved binding in WT form”; “# Peptides with improved binding in Omicron form”) (upper table).
