# Supplementary material for: Vaccines against the original strain of SARS-CoV-2 provide T cell memory to the B.1.1.529 variant
Source: Commun Med (Lond). 2022 Nov 2;2:140. doi: 10.1038/s43856-022-00203-7 (PMC9629766; doi:10.1038/s43856-022-00203-7)
Supplement: Supplementary file 5 — Reporting Summary [file 43856_2022_203_MOESM5_ESM.pdf]

nature research

Corresponding author(s): DBPR COMMSMED-22-0071-T

Last updated by author(s): Sep 29, 2022

Reporting Summary

Nature Research wishes to improve the reproducibility of the work that we publish. This form provides structure for consistency and transparency in reporting. For further information on Nature Research policies, see our Editorial Policies and the Editorial Policy Checklist.

Statistics

For all statistical analyses, confirm that the following items are present in the figure legend, table legend, main text, or Methods section.

n/a	Confirmed	
☐	☒	The exact sample size (*n*) for each experimental group/condition, given as a discrete number and unit of measurement
☐	☒	A statement on whether measurements were taken from distinct samples or whether the same sample was measured repeatedly
☐	☒	The statistical test(s) used AND whether they are one- or two-sided<br>*Only common tests should be described solely by name; describe more complex techniques in the Methods section.*
☒	☐	A description of all covariates tested
☐	☒	A description of any assumptions or corrections, such as tests of normality and adjustment for multiple comparisons
☐	☒	A full description of the statistical parameters including central tendency (e.g. means) or other basic estimates (e.g. regression coefficient) AND variation (e.g. standard deviation) or associated estimates of uncertainty (e.g. confidence intervals)
☒	☐	For null hypothesis testing, the test statistic (e.g. *F*, *t*, *r*) with confidence intervals, effect sizes, degrees of freedom and *P* value noted<br>*Give P values as exact values whenever suitable.*
☒	☐	For Bayesian analysis, information on the choice of priors and Markov chain Monte Carlo settings
☒	☐	For hierarchical and complex designs, identification of the appropriate level for tests and full reporting of outcomes
☒	☐	Estimates of effect sizes (e.g. Cohen's *d*, Pearson's *r*), indicating how they were calculated

Our web collection on statistics for biologists contains articles on many of the points above.

Software and code

Policy information about availability of computer code

Data collection	*Provide a description of all commercial, open source and custom code used to collect the data in this study, specifying the version used OR state that no software was used.*
Data analysis	NetMHCpan-algorithm (https://services.healthtech.dtu.dk/service.php?NetMHCpan-4.1)<br>MHC class II-directed predictions (https://services.healthtech.dtu.dk/service.php?NetMHCIIpan-4.0)

For manuscripts utilizing custom algorithms or software that are central to the research but not yet described in published literature, software must be made available to editors and reviewers. We strongly encourage code deposition in a community repository (e.g. GitHub). See the Nature Research guidelines for submitting code & software for further information.

Data

Policy information about availability of data

All manuscripts must include a data availability statement. This statement should provide the following information, where applicable:

- Accession codes, unique identifiers, or web links for publicly available datasets
- A list of figures that have associated raw data
- A description of any restrictions on data availability

The data generated during and/or analyzed during the current study are available from the corresponding author on reasonable request and require permission of the study participants.The data generated during and/or analyzed during the current study are available from the corresponding author on reasonable request and require permission of the study participants. Supplementary Data 1 contains the data underlying Figures 1 and 2.

Field-specific reporting

Please select the one below that is the best fit for your research. If you are not sure, read the appropriate sections before making your selection.

☒ Life sciences ☐ Behavioural & social sciences ☐ Ecological, evolutionary & environmental sciences

For a reference copy of the document with all sections, see nature.com/documents/nr-reporting-summary-flat.pdf

Life sciences study design

All studies must disclose on these points even when the disclosure is negative.

Sample size	A sample size was not calculated before the study. The number of samples was the result of the number of available participants in the study.
Data exclusions	No data were excluded.
Replication	The assay used for the study was tested for reproducibility and repeatability in the team. Data cannot be replicated since the timepoint post vaccination for the analysis is in the past.
Randomization	Covariants were not controlled, since numbers of 2- and 3-times vaccinated individuals was limited. No further randomisation was done.
Blinding	Analysts were blinded in terms of the vaccination status of the study stubjects.

Reporting for specific materials, systems and methods

We require information from authors about some types of materials, experimental systems and methods used in many studies. Here, indicate whether each material, system or method listed is relevant to your study. If you are not sure if a list item applies to your research, read the appropriate section before selecting a response.

Materials & experimental systems

n/a	Involved in the study	
☐	☒	Antibodies
☒	☐	Eukaryotic cell lines
☒	☐	Palaeontology and archaeology
☒	☐	Animals and other organisms
☐	☒	Human research participants
☐	☒	Clinical data
☒	☐	Dual use research of concern

Methods

n/a	Involved in the study	
☒	☐	ChIP-seq
☐	☒	Flow cytometry
☒	☐	MRI-based neuroimaging

Antibodies

Antibodies used	anti-CD3 – APC (Miltenyi Biotec, Cat. No. 130-113-135), anti-CD14 – VioBlue (Miltenyi Biotec, Cat. No.130-110-525), anti-CD20 – VioBlue (Miltenyi Biotec, Cat. No.130-111-531), anti-CD4 – VioBright515 (Miltenyi Biotec, Cat. No.130-114-535), anti-CD8 – VioGreen (Miltenyi Biotec, Cat. No.130-110-684), anti-IFN-γ – PE (Miltenyi Biotec, Cat. No.130-113-496), anti-TNF-α – PEVio770 (Miltenyi Biotec, Cat. No.130-120-492), anti-CD154 – APCVio770 (Miltenyi Biotec, Cat. No.130-114-130), and anti-IL-2 – PEVio615 (Miltenyi Biotec, Cat. No.130-111-307)
Validation	See manufacturer´s Website: https://www.miltenyibiotec.com

Human research participants

Policy information about studies involving human research participants

Population characteristics	See manuscript.
Recruitment	Study participants were selected on the basis of the vaccination status: 2-times vs. 3-times vaccinated individuals.
Ethics oversight	Medical association of North Rhine, germany.

Note that full information on the approval of the study protocol must also be provided in the manuscript.

Clinical data

Policy information about clinical studies

All manuscripts should comply with the ICMJE guidelines for publication of clinical research and a completed CONSORT checklist must be included with all submissions.

Clinical trial registration	ID151/2020
Study protocol	Available via Medical association of North Rhine, ID151/2020 or via the corresponding author.
Data collection	Data were collected internally at Miltenyi Biotec according to the study protocol.
Outcomes	Outcomes were not pre-defined.

Flow Cytometry

Plots

Confirm that:

☐ The axis labels state the marker and fluorochrome used (e.g. CD4-FITC).

☐ The axis scales are clearly visible. Include numbers along axes only for bottom left plot of group (a 'group' is an analysis of identical markers).

☒ All plots are contour plots with outliers or pseudocolor plots.

☐ A numerical value for number of cells or percentage (with statistics) is provided.

Methodology

Sample preparation	To assess the reactivity of SARS-CoV-2 specific T cells, PBMC from all whole blood samples were isolated via density-gradient centrifugation using Pancoll® (Pan Biotech, Aidenbach, Germany, Cat. No. P04-60500), according to the manufacturer's protocol using CliniMACS® PBS/EDTA Buffer (Miltenyi Biotec, Bergisch Gladbach, Germany, Cat. No. 200-070-025). To remove remaining thrombocytes from isolated PBMCs, samples were washed twice by resuspending them in 50 mL CliniMACS® PBS/EDTA buffer and subsequently centrifuged at 200 g for 15 min. Afterwards, cell numbers were determined using a Sysmex XP-300 device (Sysmex, Norderstedt, Germany). Cells were then plated out on a 96-well flat-bottom plate (Falcon, New York, USA, Cat. No. 353072) at a concentration of 1E6 cells/0.1 mL RPMI-1640 Medium (Biowest, Nuaillé, France, Cat. No. L0501-500) supplemented with 5% human AB Serum (Capricorn, Ebsdorfergrund, Germany, Cat. No. HUM-3B, Lot. CP20-3472) and 1x Gibco Anti-Anti (Thermo Fisher Scientific, Waltham, USA, Cat. No. 11580486) /well. Next, SARS-CoV-2 reactive T lymphocytes were stimulated by adding 1 µg/mL SARS-CoV-2-derived peptides from A) a pool of 83 15mer-peptides covering all mutations of the B.1.1.529 strain (“B1.1.529 Mutation Pool”, Miltenyi Biotec, Cat. No. 130-129-928), B) a pool of 83 15mer-, reference-peptides to A derived from the wildtype virus (“WT Reference Pool”, Miltenyi Biotec, Cat. No. 130-129-927), or C) a mega-pool of 360 15mer-peptides covering the complete sequence of the wildtype spike-protein (“Prot_S Complete”, Miltenyi Biotec, Cat. No. 130-127-951). An unstimulated control sample was prepared as negative control. The cells were then incubated for 6 hours at 37°C, 5% CO2. After 2 hours of incubation, 2 µg/mL Brefeldin A (Sigma-Aldrich, St. Louis, USA, Cat. No. B7651) was added to each well. Reactivities of CD4 and CD8 T cell subsets after stimulation with SARS-CoV-2 derived peptides were quantified by the staining of the activation-associated marker CD154, together with staining of intracellular cytokines IFN-γ, TNF-α, and IL-2. This staining was performed in a 96-well V-bottom plate (Sigma-Aldrich, St. Louis, USA, Cat. No. Z667234) into which cells were transferred after adding 100 µl PBS/EDTA (2mM) buffer to each sample. After centrifugation at 300 g for 5min the supernatant was discarded and dead cells were stained with ViobilityTM 450/452 Fixable Dyes (Miltenyi Biotec, Cat. 130-109-816) according to the manufacturer’s instructions. Afterward, cells were washed with PBS, centrifuged at 300 g for 5 min, and the supernatant was discarded, followed by the fixation of cells using Inside Fix (Inside Stain Kit, Miltenyi Biotec, Cat. No. 130-090-477), according to the manufacturer’s instruction. Afterwards, cells were permeabilized by resuspending and centrifuging them in 100µl Inside Perm (Inside Stain Kit, Miltenyi Biotec, Cat.-No. 130-090-477), using the described settings. Finally, cells were stained using the following antibody-cocktail: anti-CD3 – APC (Miltenyi Biotec, Cat. No. 130-113-135), anti-CD14 – VioBlue (Miltenyi Biotec, Cat. No.130-110-525), anti-CD20 – VioBlue (Miltenyi Biotec, Cat. No.130-111-531), anti-CD4 – VioBright515 (Miltenyi Biotec, Cat. No.130-114-535), anti-CD8 – VioGreen (Miltenyi Biotec, Cat. No.130-110-684), anti-IFN-γ – PE (Miltenyi Biotec, Cat. No.130-113-496), anti-TNF-α – PEVio770 (Miltenyi Biotec, Cat. No.130-120-492), anti-CD154 – APCVio770 (Miltenyi Biotec, Cat. No.130-114-130), and anti-IL-2 – PEVio615 (Miltenyi Biotec, Cat. No.130-111-307). All antibodies were used in a 1:50 dilution. Staining was done according to the manufacturer’s instructions Cells were washed by addition of Inside Perm and centrifugation at 300 g for 5 min. After discarding of the supernatant cells were resuspended in PBS/EDTA/BSA – buffer for subsequent flow-cytometric analysis.
Instrument	Data acquisition was done using MACSQuant16 – flow cytometer (Miltenyi Biotec, Cat. No. 130-109-803).
Software	MACS Quantify. FlowJo10.7.2
Cell population abundance	See manuscript Materials & Methods section.
Gating strategy	Lymphocyte -> Doublet Exclusion ->CD3+CD14-CD20- -> CD4+ / CD8+ -> Activation markers as indicated in the manuscript.

☒ Tick this box to confirm that a figure exemplifying the gating strategy is provided in the Supplementary Information.
